# CACNA1S Arg528Cys mutation in a young Chinese man with thyrotoxic hypokalemic periodic paralysis

**DOI:** 10.1002/ccr3.3054

**Published:** 2020-07-01

**Authors:** Nader Rezkalla, Kamran Imam, Miriam Marti, Karen Ip, Ardavan Mashhadian, Antonio Liu

**Affiliations:** ^1^ Ross University School of Medicine Miramar Florida USA; ^2^ Department of Neurology Adventist Health White Memorial Los Angeles California USA; ^3^ Department of Nephrology California Hospital Medical Center Los Angeles California USA; ^4^ Department of Neurology California Hospital Medical Center Los Angeles California USA

**Keywords:** endocrinology, genetics, metabolic disorders, neurology

## Abstract

It has long been believed that the patients with thyrotoxic hypokalemic periodic paralysis (THPP) may harbor genetic mutations commonly found in familial hypokalemic periodic paralysis. Despite extensive testing, such a mutation has escaped detection until now.

## Graphical abstract

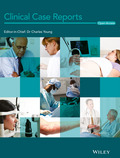

## 1 INTRODUCTION

Hypokalemic periodic paralysis (HypoPP) is a rare neurological disorder characterized by episodic disabling weakness that is associated with a low serum potassium level. Patients often recover rapidly if serum potassium is repleted. The majority of patients are asymptomatic in between attacks. The disease is often broken down into three main types: familial (FPP), thyrotoxic (THPP), and sporadic (SPP).

Familial periodic paralysis (FPP) has been found to arise from mutations in genes that encode sodium channels (*SCN4A*), potassium channels (*KCNE3*), and calcium channels (*CACNA1S*). *CACNA1S* is the gene that encodes the L‐type calcium channel α1‐subunit.^1^, ^2^ The mutations occur due to single nucleotide polymorphisms, which refer to the process of multiple alleles occupying the same locus within a specified population.

Thyrotoxic hypokalemic periodic paralysis (THPP) has been described to be common in the Chinese population.^3^ Cases of THPP can be caused by thyrotoxicosis. The higher circulating levels of hormones in thyrotoxicosis including thyroxin, androgens, and insulin increase the activity of Na^+^/K^+^‐ATPase. The increased activity leads to disrupted ion channels, intracellular potassium uptake with subsequent hypokalemia, hyperpolarization of cells, and lack of calcium conductance of skeletal muscular cells that ultimately leads to paralysis. A large carbohydrate meal can cause an influx of insulin that further worsens the paralysis, as was seen in our patient. Although plasma potassium concentration is low, body potassium stores are typically normal in THPP. Attempting to resolve the paralysis by administering potassium supplementation usually leads to rapid release of potassium from cells, which can lead to rebound hyperkalemia. Early diagnosis aids in definitive management as nonselective beta blockers can be used to treat both hyperthyroidism and hypokalemia, while also preventing the risk of rebound hyperkalemia.^4^

## 2 CASE PRESENTATION

A 23‐year‐old graduate school student from China presented to the emergency department with 2‐day history of progressively worsening bilateral lower extremity painless weakness. The patient was unable to ambulate without assistance. Six months ago, he had an episode of mild weakness that resolved spontaneously over the course of a few days, for which he did not seek out medical care. The patient denied any past medical history and stated that none of his family members had ever exhibited similar symptoms. He was not adopted, and he is well‐acquainted with his extended family in Southern China. He denied smoking, alcohol use, and illicit drug use. He endorsed having a large carbohydrate meal consisting of rice prior to admission, and admitted to regularly binge drinking soda. He denied complaints of weight loss, and heat or cold intolerance. His school performance had not been affected.

On physical examination, vital signs revealed a temperature of 36.9°C and the patient remained afebrile throughout hospitalization. He appeared to be in no respiratory distress. At the time of examination, weight was 70 kg, height was 180 cm, and BMI was 19.8. Blood pressure was 130/89, heart rate was 90 bpm, and O_2_ Sat was 99% on room air. The patient appeared to be a well‐built young man. He was oriented x4. Cranial nerve examination was unremarkable, and he had no complaints of dysphagia or dysarthria. Extraocular movements were all intact without fatigability. Thyroid examination was normal to examination and ultrasound. There was no exophthalmos or goiter. Motor examination showed normal tone and bulk, and strength was decreased symmetrically: 4+/5 in his upper extremities and 3/5 in his lower extremities. All reflexes were 2‐/4, clonus absent, and Babinski sign was negative. Sensory examination showed an intact light touch, proprioception, and ice sensation. The patient was unable to ambulate, but denied fecal or urinary incontinence. EKG was unremarkable.

CBC was within normal limits. WBC 9800 (5000‐11000), platelet count 308 000 (140 000‐400 000), hemoglobin‐hematocrit 15 g/dL/44% (12‐16/37‐47), creatine kinase 350 U/L (30‐200), and troponin I < 0.01 ng/mL (<0.29). Laboratory examination showed K^+^ 2.0 mmol/L (3.4‐5.0), TSH < 0.01 mU/L (0.35‐4.94), and FT4 2.17 ng/dL (0.8‐1.4). Urine toxicology was negative. MRI of the head and whole spine was negative.

During hospitalization, the patient's motor strength did not worsen. He experienced rapid improvement over 36 hours following potassium replacement. Due to suspicion, hypokalemic and hyperkalemic periodic paralysis 6‐gene panel test was performed through Fulgent Genetics Laboratory. Gene sequencing with deletion and duplication analysis eventually revealed a heterozygous Arg528Cys mutation (c.1582C > T) at *CACNA1S*. Hyperthyroidism was worked up, however the patient left against medical advice. He was advised to follow up with his primary doctor to ensure reaching a euthyroid state. The patient was recommended to improve his diet, and be placed on propranolol to ensure that no further episodes of thyrotoxicosis recur.

## 3 DISCUSSION

As per the literature review, research and data collection of HypoPP began in the late 1960s. All articles from that era focused on the clinical features and management. As per our search, genetic dissection of the various forms of HypoPP started in 1994. Mutations were found in the L‐type voltage‐gated calcium channel encoded by *CACNA1S* (also named *CACNL1A3*),^5^, ^6^ revealing the first association with HypoPP. Sequencing exhibited a guanine to adenosine base exchange at nucleotide 1583 (c.1583G > A), causing a missense mutation (arginine to histidine substitution) at the 528 residue (Arg528His) α1‐subunit of the calcium channel. It was proven to be associated with FPP.^7^, ^8^ Further study found FPP patients harboring mutations in *SCN4A*, *KCNE3*, *KCNJ2*, and *CACNA1S*. In the *CACNA1S* variant, histidine substitutions are the most common at the 528 residue.^2^

Based on similar clinical features, many scholars, clinicians, and scientists attempted to find FPP mutations in THPP and SPP patients. However, most studies found no affiliation initially. In 2002, Dias da Silva et al found that mutations associated with FPP in the calcium channel α1 subunit gene are not linked to THPP. They did, however, find that polymorphisms within Ca_v_1.1 were more common among THPP patients and represented a novel finding.^9^ Attempts to find links to THPP through mutations in the *CACNA1S* gene were also fruitless, although there seemed to be further indications that there were associated polymorphisms.^10^, ^11^, ^12^, ^13^

Well into the 21st century, cases emerged that found actual mutations associated with THPP. In 2004, Lane et al found that the genetic spectrum of FPP and THPP seems to overlap in the sodium channel gene, *SCN4A*. They identified an Arg672Ser mutation that they connected to THPP.^14^ Studies reported in 2010 and 2013 prove that mutations in the potassium channel gene, *KCNJ18*, encoding K_ir_2.6 cause susceptibility to THPP.^15^, ^16^ While there have been reported cases associating THPP with mutations, extensive research has shown that none have been mutations substituting cysteine for arginine at the 528 residue (Arg528Cys) in *CACNA1S*.^17^, ^18^, ^19^

Despite case reports and large‐scale studies in the past 2 decades, so far, no mutation at *CACNA1S*, commonly found in FPP, has been identified to be associated with THPP. As per our search, there has only been one documented patient in 2014 that appeared to have the exact mutation at the same location. That patient, however, suffered from adrenal hyperplasia and was reported to have a high TSH with no signs of thyrotoxicosis.^20^ Further research studies are needed in the *CACNA1S* gene and the specific pathophysiology that accounts for the different clinical manifestations seen from mutations in this gene.

Therefore, we conclude that we have reported a mutation never documented before—a genetic defect commonly associated with FPP (c.1582C > T p.Arg528Cys in *CACNA1S*)—in a young Chinese male patient with THPP having no known family history of periodic paralysis.

## CONFLICT OF INTEREST

None declared.

## AUTHOR CONTRIBUTIONS

All the authors: made substantial contribution to the preparation of this manuscript and approved the final version for submission. NR: is a first author and drafted initial manuscript. AL: is a principal investigator and supervising physician in charge of managing patient's paralysis. AM: is a supervising physician in charge of managing the patient's thyrotoxicosis and hypokalemia. NR, KI, MM, and KI: revised the manuscript for critically important intellectual content and worked under supervising physicians.
